# Optical Coherence Tomography—OCT for Characterization of Non-Atherosclerotic Coronary Lesions in Acute Coronary Syndromes

**DOI:** 10.3390/jcm11010265

**Published:** 2022-01-05

**Authors:** Mihail Spînu, Laurenţiu Horea Onea, Călin Homorodean, Maria Olinic, Mihai Claudiu Ober, Dan Mircea Olinic

**Affiliations:** 1 Medical Clinic Number 1, “Iuliu Haţieganu” University of Medicine and Pharmacy, 400006 Cluj-Napoca, Romania; spinumihail04@gmail.com (M.S.); onea.lau@gmail.com (L.H.O.); mariaolinic2003@gmail.com (M.O.); danolinic@gmail.com (D.M.O.); 2 Department of Interventional Cardiology, Cluj County Emergency Hospital, 400006 Cluj-Napoca, Romania; mober2009@gmail.com

**Keywords:** non-atherosclerotic coronary lesions, spontaneous coronary artery dissection, spontaneous recanalization of coronary thrombus, acute coronary syndromes, optical coherence tomography

## Abstract

Cardiovascular diseases are the main cause of death worldwide, with coronary artery disease being the predominant underlying etiology. The most prevalent coronary lesions are represented by the atherosclerotic plaques, in more than 85% of cases, but there are several other non-atherosclerotic lesions such as spontaneous coronary artery dissection and/or hematoma and spontaneous recanalization of coronary thrombus, which are less common, approximately 5% of cases, but with similar clinical manifestations as well as complications. There are insufficient data regarding the pathological mechanism, true prevalence and optimal treatment of these kind of coronary lesions. Optical coherence tomography (OCT) is an intracoronary imaging technique, developed in order to overcome the diagnostic limitations of a standard coronary angiography and has an extremely high resolution, similar to that of a usual histological evaluation of a biopsy sample, thus, OCT provides a histological-like information, but in a in vivo environment. The aim of this article is to review the current knowledge regarding non-atherosclerotic coronary lesions, with an emphasis on the importance of OCT for optimal identification, characterization of pathogenic mechanisms and optimal treatment selection.

## 1. Introduction

Cardiovascular diseases represent a major health burden, with coronary artery disease (CAD) as the most important cause of morbidity and mortality [1]. Every effort is made for improving CAD diagnosis, identifying patients with an indication for revascularization and optimal revascularization treatment through either interventional cardiology or cardiac surgery, resulting in a progressive decrease in CAD mortality [2]. Coronary angiography (CA) is the gold standard invasive procedure currently used for CAD diagnosis, decision-taking and assessing efficacy of percutaneous coronary interventions (PCIs) [2,3]. The main limitations of CA imaging are the two-dimensional (2D) view of the three-dimensional (3D) coronary artery lumen and wall structure, as well as, in some cases, the poor accuracy of estimating atherosclerotic plaque volume, morphology and degree of stenosis severity [2,3].

Optical coherence tomography (OCT) further improves invasive intracoronary imaging, offering the third dimension, and with that, new insights on the coronary wall and lumen, due to its high resolution (10 μm), similar to that of a usual histological evaluation of a biopsy sample and is considered to be an “optical biopsy” that provides in vivo imaging [2,3,4]. OCT provides accurate characterization of atherosclerotic plaques, in terms of severity and extension, and identifies high-risk and complicated plaques, as well as coronary thrombi [2,3,4]. OCT is therefore not only a very well recognized in vivo CAD research tool [5,6], but also a method to be used safely, in everyday practice, as a complement to CA, due to progressive and continuous improving of OCT devices and software over the last ten years [3,7].

The most prevalent coronary lesions are represented by the atherosclerotic plaques, in more than 85% of cases [1], but there are several other non-atherosclerotic lesions such as spontaneous coronary artery dissection and/or hematoma [8,9] and spontaneous recanalization of coronary thrombus [10,11], which are less common, approximately 5% of cases [1], but with similar clinical manifestations as well as complications. There are insufficient data regarding the pathological mechanism, true prevalence and optimal treatment of these kind of coronary lesions.

The aim of this article is to review the current knowledge regarding all non-atherosclerotic coronary lesions, with an emphasis on the importance of OCT for optimal identification, characterization of pathogenic mechanisms and optimal treatment selection.

## 2. OCT Principles

At the origin, OCT was used for the 2D imaging of the retina, using optical one-dimensional low coherence reflectometry [7]. After the addition of transverse scanning (B-scan) in 1991, OCT technique was applied in various medical and non-medical settings [12].

OCT uses a near-infrared light source with a 1300 nm frequency. This wavelength allows a tissue penetration of up to 3 mm. Higher light wavelengths allow deeper tissue penetration, but there are also other more important characteristics, such as tissue absorption and the refractive surface capacity that defines ideal frequency [12].

During OCT evolution, two systems were used: The time-domain (TD)-OCT and the frequency/Fourier domain (FD)-OCT techniques [13].

Erythrocytes have a high iron concentration and represent an important source of artifacts, therefore, in order to perform OCT, there is a need for emptying the coronary vessel [13]. This process is different in the two OCT systems.

OCT images are generated in a similar manner to echo techniques, using two arms of the light bean: a reference arm and a tissue sweeping one. The reflected, backscattered waves from different tissue depths have different time delays as compared to the reference wave. Due to the high velocity of light (much higher as compared to ultrasounds), OCT needs an interferometer in order to assess the reflective bean time delays and amplitude changes induced by the tissue optical characteristics. The reference wave is generated using a reflecting mirror and combined with the tissue reflected wave in the interferometer. The first-generation OCT system, TD-OCT, uses a mechanically moving mirror. Due to the limitations of the movement speed of the mirror, the image generation time is increased. Therefore, TD-OCT requires an over-the-wire low-pressure occlusion balloon in order to selectively displace the blood during acquisition. However, this process involves an adjacent procedural risk, related to myocardial ischemia, vascular perforation, arrhythmias and death [14].

The second-generation OCT system FD-OCT employs a fixed mirror and a narrow bandwidth light source that sweeps rapidly between different wavelengths (from 1250 to 1350 nm). It generates interference patterns at all these wavelengths and through Fourier transformations processing it provides amplitude profile and time delay of the waves reflected from different depths [15]. All echo time delays along one A-line (one axial line of a frame) are measured at the same time by the FD detector. Thus, it allows better signal to-noise ratio and faster imaging processing time [16]. Image acquisition is performed during a normal selective contrast bolus injection. Procedural complications are thereby reduced [17].

The current FD-OCT systems allow acquisition of 100,000 axial lines per second generating up to 200 frames per second (each consisting of 500–1000 lines). Therefore, a pullback speed of 40 mm/s is possible with pullback lengths up to 150 mm with a distance between frames of 0.1–0.25 mm [15].

OCT is used in current clinical practice immediately after CA to assess coronary lesions, establish indications for PCI, choose strategy and guide PCI. After performing PCI, OCT is used to evaluate the immediate result, optimize it, and in evolution, identify stent-failure mechanisms [3].

### OCT Limitations and Disadvantages

OCT increases operating times, as well as contract volume agent. There is also a slight increase in procedural complication, similar to PCI procedures. OCT cannot be used in cases of occluded vessels or clinical unstable patients. These disadvantages are overwhelmed by the net advantages offered by OCT use in interventional cardiology, by providing the correct diagnosis and, respectively, a better procedural and clinical outcome.

## 3. Spontaneous Coronary Artery Dissection (SCAD)

### 3.1. Definition

SCAD is defined as a nontraumatic, noniatrogenic, and nonatherosclerotic separation of the coronary arterial wall by an intramural hematoma (IMH), creating a false lumen, which then compresses the true lumen, causing myocardial ischemia or infarction [18].

First reported in 1931, SCAD is a very rare cause of acute coronary syndrome (ACS) [19] It is predominantly present in women, with the majority of patients being diagnosed at autopsy. Most patients are under 50 years old, with the mean age of presentation being 35 to 40 years [20].

### 3.2. Epidemiology

The true incidence and prevalence of SCAD is unknown, due to an important under/misdiagnosis. Recent publications show that SCAD was diagnosed on coronary angiography in up to 4% of all acute coronary syndromes (ACS) and in 0.5% of autopsy reports, as the cause for sudden cardiac death. The most affected gender is by far the female sex, in a 9:1 ratio, especially women with few or no traditional cardiovascular risk factors. Up to 35% of MI in women younger than 50 years old and 43% of the pregnancy-associated MI are caused by SCAD. SCAD can affect every age group, but the most prevalent is the 4th and 5th decade [21].

### 3.3. Etiology

**Figure 1:**
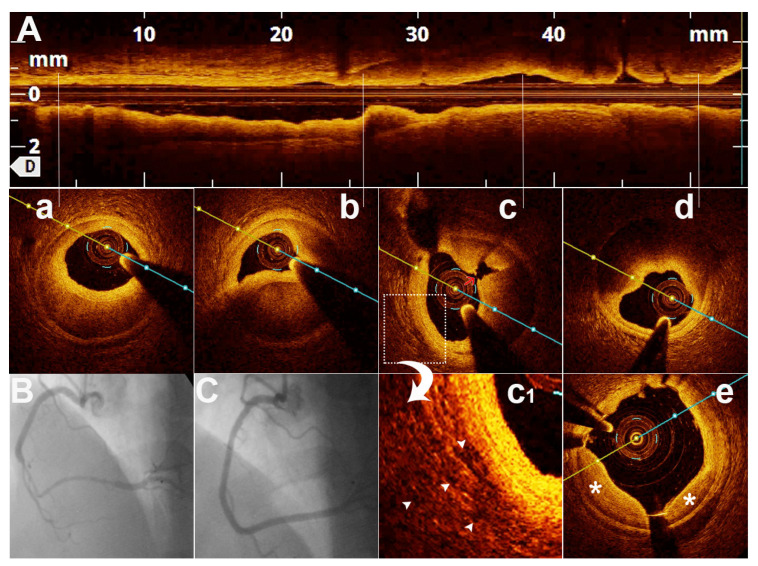
SCAD in a 47 years old female patient with inferior ST segment elevated myocardial infarction. (**A**)—OCT—longitudinal view—SCAD—true and false lumen; (**B**)—coronary angiography of SCAD; (**C**)—post PCI result with 2 drug eluting stents; (**a**–**d**)—OCT—transversal views—SCAD—true and false lumen; (**c**)—SCAD fenestration (red arrow); (**c1**)—rich vasa-vasorum network (white arrowheads); (**e**)—OCT—post PCI result prolapse of intimal flap between stent struts (asterisk).

**Figure 2:**
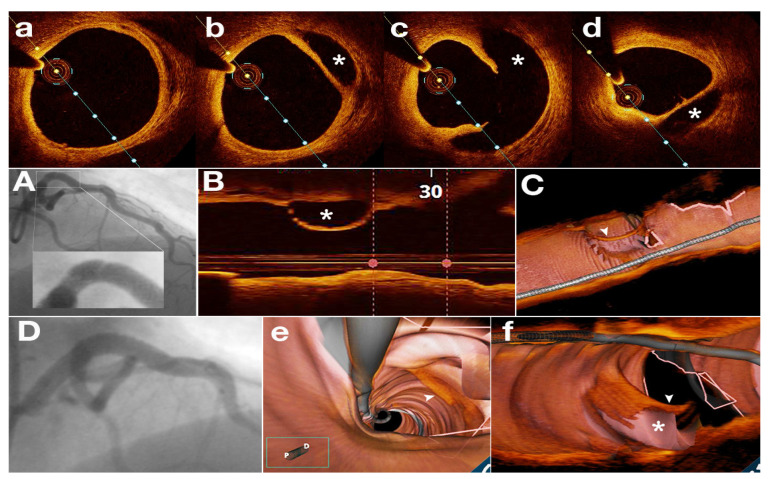
SCAD in a 46 years old female patient with anterior ST segment elevated myocardial infarction. (**A**)—coronary angiography of SCAD on proximal left anterior descending artery; (**B**)—OCT—longitudinal view—SCAD—true and false lumen (asterisk); (**C**)—OCT—3D reconstruction, dissection flap—white arrowhead; (**D**)—coronary angiography—post PCI result with one drug eluting stent; (**a**–**d**)—OCT—transversal views—SCAD—true and false lumen (asterisk); (**e**,**f**)—OCT—endoscopic view (dissection flap—white arrowhead, false lumen—asterisk).

**Figure 3:**
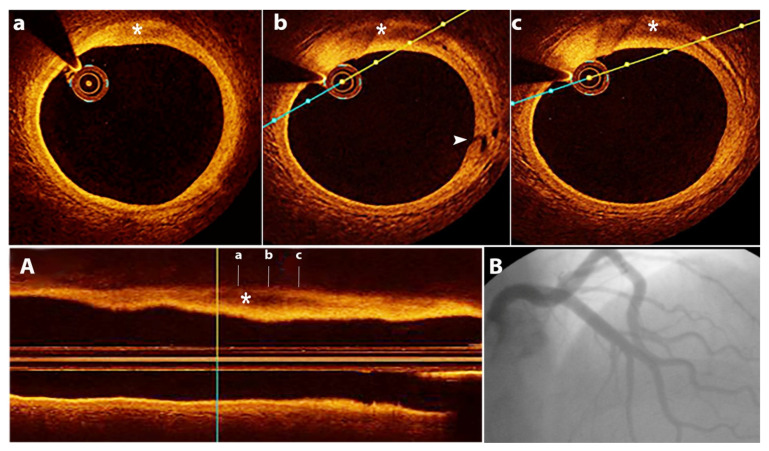
SCAD in a 41 years old male patient with anterior ST segment elevated myocardial infarction. (**A**)—OCT—longitudinal view—in wall hematoma (asterisk); (**B**)—normal coronary arteries on angiography; (**a**–**c**)—OCT—transversal views—SCAD—in wall hematoma (asterisk)—dissection entry point (white arrowhead in (**b**)).

**Figure 4:**
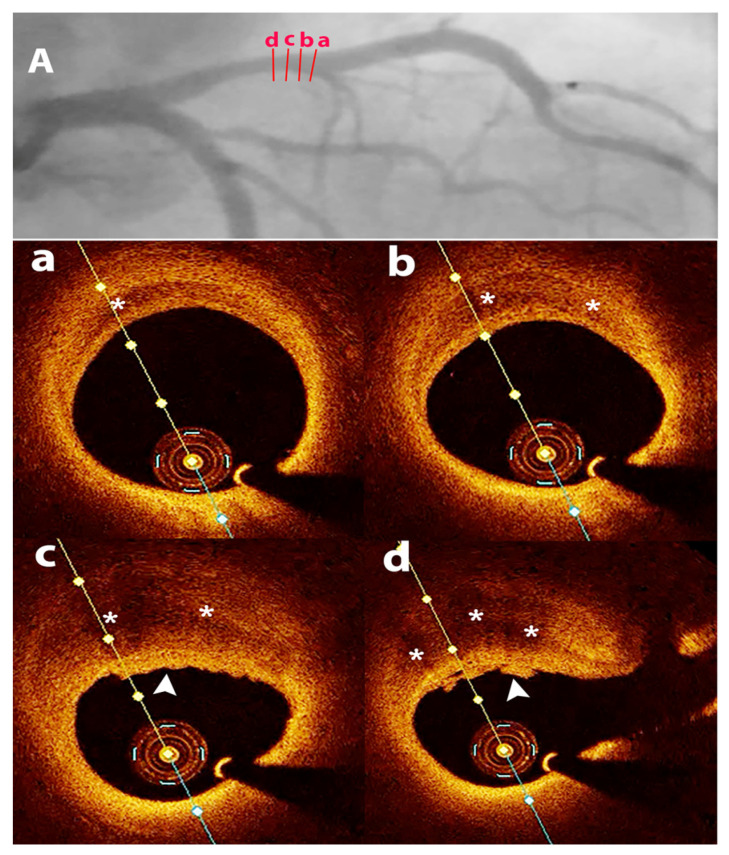
SCAD in a 48 years old female patient with non ST segment elevation myocardial infarction. (**A**)—coronary angiography—borderline proximal left anterior descending artery stenosis; (**a**–**d**)—OCT—transversal views—SCAD—in wall hematoma (asterisks) with thrombus on the intimal surface (white arrowhead in (**c**,**d**)), with significant lumen reduction, followed by PCI.

The pathophysiology of SCAD remains incompletely understood, but there is often an underlying arteriopathy that weakens the arterial wall, with associated additional physical, emotional, and/or hormonal stressors that trigger the dissection [18]. The separation can occur between the intima, media, or adventitia and can originate from an intimal tear (Figure 1 and Figure 2) leading to dissection into the arterial wall or may result from spontaneous bleeding from ruptured vasa vasorum without intimal tear (Figure 3 and Figure 4) [18].

Some authors suggest that atherosclerosis is the main etiology in about 30% of patients with SCAD [21,22,23,24], due to an increased density of the vasa vasorum, that leads to bleeding and dissection of the coronary artery [23].

Another important condition associated with SCAD represents the peripartum stage. Due to exposure to recurrent and chronic hormonal pregnancy changes with elevated cardiac output, increased total blood volume, and straining forces during labor, may result in increased wall stress and, therefore, SCAD appearance [25].

**Figure 5:**
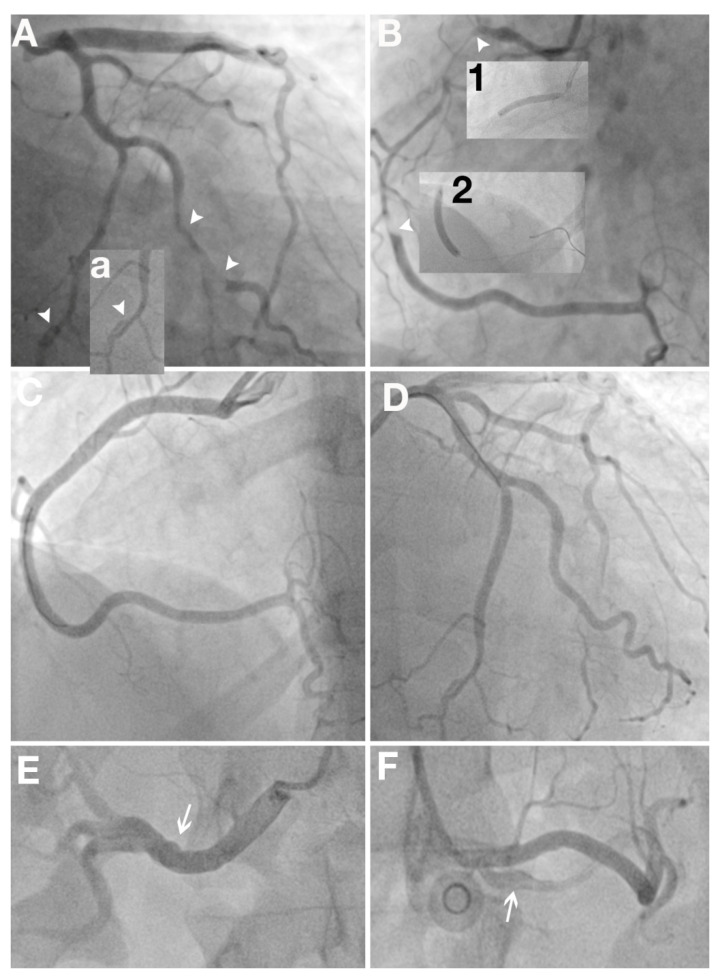
Multivessel SCAD in a 45 years old female patient with inferior ST segment elevated myocardial infarction. (**A**)—coronary angiography—SCAD (white arrowheads) on left circumflex artery (**a**) and obtuse marginal artery (OM); (**B**)—coronary angiography—long SCAD (white arrowheads) on righ coronary artery (RCA); (**1**,**2**)—SCAD PCI strategy—distal and proximal stent implantation to stop progression of the dissection; (**C**,**D**)—very good post PCI result on RCA and OM; (**E**,**F**)—right and left renal arteries with FMD aspect (white arrows). Due to continuous intense angina, we did not perform OCT.

In the majority of cases, however, an underlying condition which may lead to SCAD cannot be identified. Other reports suggest that autoimmune and genetic connective tissue diseases, fibromuscular dysplasia (FMD), and other non-medical conditions such as vigorous exercise, cocaine abuse and prolonged sneezing can also cause SCAD (Figure 5) [20].

### 3.4. Clinical Presentation

SCAD patients usually present with chest pain in the setting of ACS, but, also, as a complication of ACS—malignant arrhythmias, cardiogenic shock and sudden cardiac death. Symptoms of acute heart failure due to reduced left ventricular ejection fraction can also be encountered [21].

### 3.5. Diagnosis of SCAD

The main imaging method for SCAD diagnosis is standard coronary angiography (CA). There is an angiographic classification [26] which describes three types of SCAD: Type 1 pathognomonic appearance of multiple radiolucent lumen; Type 2 describes diffuse smooth stenosis of varying length (typically > 20 mm) and severity, with type A having normal artery segments proximally and distally to SCAD and type B having diffuse narrowing extending to the apical tip of the artery [26]; Type 3 describes a focal, less than 20 mm stenosis that mimics atherosclerosis [26].

Type 2 CA aspect of SCAD is the most encountered in clinical practice and, usually, are observed in the mid to distal part of the vessel [21]. The most affected artery is the left anterior descending one, but, in up to one quarter of cases, there are multiple arteries involved [21].

#### 3.5.1. Intravascular Imaging

OCT or Intravascular Ultrasound (IVUS) offers the third dimension to the coronary lumen and wall and, due to CA limitations and low sensitivity, is the only method for accurate SCAD diagnosis [8,9,27,28,29,30]. Both methods can provide the SCAD diagnosis, but OCT, due to its superior resolution in image acquisitions, is the method of choice [8,9]. The correct diagnosis is the key for a good clinical outcome, but, in case of SCAD with indications for revascularization, OCT can also provide crucial information: presence of the coronary guide into the true lumen and identification of the stent landing zones [8,9].

#### 3.5.2. OCT in SCAD

OCT provides an extraordinary picture, in the smallest detail, of the coronary vessel. It allows the identification and measurement of all layers of the coronary wall and vascular lumen, the interface between the wall and the lumen, but also the vasa-vasorum network.

When imaging a SCAD, OCT describes key features as the true and false lumen, crescent shape of the false lumen, the presence of fenestrations between the two lumens, adventitial microvessels (vasa vasorum) as well as pathological features of the undissected segments [31].

OCT provides insights into SCAD mechanisms: intimal tear with blood accumulating in the media, or wall hematoma due to micro-vessel rupture. Identification of fenestrations indicates the intimal tear as the possible cause. However, fenestrations are often not detected, supporting the role of wall hemorrhage in the dissection mechanism. In non-fenestrated SCAD, there were indirect signs of false lumen pressurization with external elastic lamina expansion and increased false lumen area [32]. These data suggest that most SCADs might be the result of hemorrhage, producing fenestrations only when the internal pressure is too high [32].

It seems logical that the source of this hemorrhage should be adventitial vasa vasorum. Nevertheless, there are contradictory OCT data regarding the number of vasa vasorum in the adventitia of patients with SCAD [31,32]. The position of the micro vessels may be more important than their number. Supporting this view, we published a case report of a coronary wall hematoma caused by a micro-vessel arising from the true lumen and tracking into the false lumen (Figure 1). Some authors suggest that increased vasa vasorum number found in some SCADs may be responsible for false lumen content resorption and not the cause of it [32].

Although it is extremely helpful for both diagnostic and therapeutic purposes, the use of OCT in the context of SCAD should be undertaken with caution because it is accompanied by a slightly increased risk of complications such as FL propagation or impaired flow in the true lumen.

#### 3.5.3. Cardiac Computed Tomography

Angiography has much lower spatial resolution compared with CA and does not allow visualization of coronary flow. It is not used as a first diagnostic modality for SCAD but is a very good option for noninvasive follow- up [33].

In cases of SCAD, screening for FMD and other extracoronary vascular abnormalities is mandatory to identify the potential cause of SCAD, and to guide management and follow-up of patients with coexistent extracoronary dissections or intracranial aneurysms. Thus, selective renal and iliac angiography for FMD and CT angiograhy for extracranial carotid, vertebral, and intracranial aneurysms are recommended [34].

### 3.6. Treatment

Natural evolution of SCAD is spontaneous healing in more than 70% of cases, thus medical therapy and inpatient observation are typically recommended as a first line treatment modality [14]. Medical therapy for SCAD is the standard medications used for coronary artery disease: beta-blockers, aspirin, renin–angiotensin system antagonists and statins [20].

### 3.7. Indications for Revascularization

Revascularization with PCI or coronary artery bypass grafting (CABG) depending on coronary, should be reserved for patients with high-risk features, such as ongoing ischemia, left main artery dissection, ventricular arrhythmias, or hemodynamic instability [8,9,20].

In cases of PCI, there are few technical aspects to be considered in order to ensure a good procedural and clinical result: the femoral access is preferred due to lower catheter manipulation, using OCT or IVUS to detect the coronary guide into the true lumen and identification of the stent landing zones, direct drug-eluting stents implantation, covering up to 10 mm on both proximal and distal edges of the IMH to avoid antegrade or retrograde propagation of the SCAD [8,9,20].

CABG is the preferred method when is an LM or multivessel SCAD and when PCI is not feasible. Some retrospective studies had shown high rates of graft failure due to SCAD healing, thus, CABG more frequently acts as bridge to vessel recovery [35].

### 3.8. Outcome and Prognosis

Despite that SCAD has a good long-term survival of more than 95%, it is associated with high rates of morbidity due to nonischemic chest pain, anxiety and depression, with adverse CV events up to 50% and with recurrent SCAD of up to 35% [14]. Importantly, pregnancy-related SCAD are associated with poorer outcomes due to LM involvement, larger size infarction and reduced LV ejection fraction [20].

## 4. Iatrogenic and Traumatic Coronary Artery Dissection

### Definition

Iatrogenic and traumatic CAD are defined as a separation of the coronary arterial wall by an IMH, creating a false lumen, which then compresses the true lumen, causing myocardial ischemia or infarction traumatic, secondary to invasive coronary procedures or to nonperforated trauma to the chest.

Iatrogenic CAD is a rare (<1%) complication of diagnostic CA, with potentially catastrophic outcomes. ICAD has a higher prevalence during coronary interventions, in relation to use of Amplatz-shaped catheters, deep intubation, unskilled manipulations and vigorous contrast ejection, especially in the presence of ulcerated plaques and variations in the coronary ostial anatomy. OCT provides the exact guidewire position and landing zones for therapeutic stent implantation, being an extremely helpful tool in managing an ICAD [2].

## 5. “Therapeutic” Coronary Dissection

This type of dissection refers to those produced after balloon angioplasty, with the purpose of predilating the coronary lesion, with the subsequent possibility of advancing the stent into the vessel. The “therapeutic” dissection produces a global deformation and fragmentation of the atheroma plaque, pushing its components towards the vascular wall. Such a dissection must be stabilized by implanting a stent, due to the extremely high thrombotic risk.

## 6. Spontaneous Recanalization of Coronary Thrombus (SRCT)

SRCT was, for the first time, observed on histopathology findings, in autopsy series, and characterized by multiple channels divided by thin septa, communicating with each other and, proximally and distally, with the normal coronary lumen [36].

The first ever in vivo identification of SRCT was performed by Terashima et al. [37] in 2002, using IVUS, describing a lotus-root appearance in the mid left anterior descending artery (LAD) of a 21 years old patient with suspected Kawasaki disease, with a history of sudden cardiac arrest at the age of one year. Since then, availability of intravascular imaging and the increasing awareness of interventional cardiologists brought to surface this pathology.

### 6.1. Epidemiology

Prevalence of SRCT lesions is believed to be around 0.1% of PCIs, reported in two different studies: 4 cases out of 4302 PCIs [10] and 33 cases out of 31,500 PCIs [38]. To date, there are published only 3 studies and 23 cases reports, accounting for a total of 66 cases [10]. In the literature, there are few different terms used to describe SCRT lesions: “honeycomb-like structure”, “lotus root structure”, “swiss cheese structure” and “spider web”. The true prevalence of SRCT might be underestimated, due to its angiographic non-specific aspect [10]. SCRT lesions has apparently a higher prevalence in men [10].

### 6.2. Etiology

**Figure 6:**
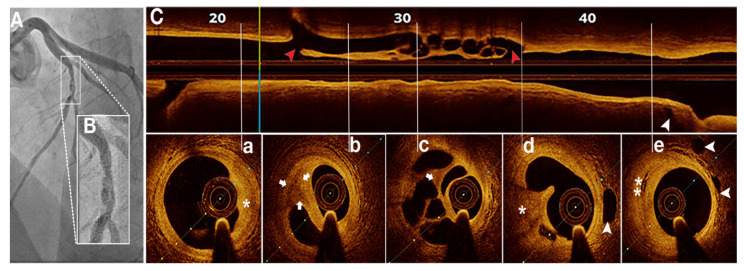
SRCT in a 43 years old female patient with unstable angina. (**A**)—coronary angiography—braided filling defect on mid left anterior descending artery; (**B**)—detailed angiographic view; (**C**)—OCT—longitudinal view of SRCT (between the red arrowheads); (**a**–**e**)—OCT—transversal views of SRCT with signs of atherosclerosis: atheroma infiltration (asterisk), hypervascularization (white arrowheads) and proteoglycan-rich tissue (white arrows). Drug eluted stent implantation was performed.

**Figure 7:**
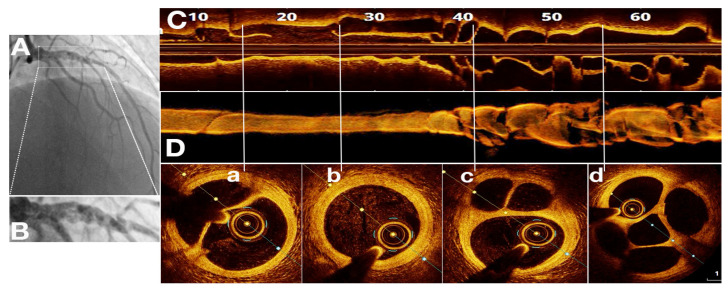
SRCT in a 41 years old male patient with anterior ST elevation myocardial infarction. (**A**)—coronary angiography—braided filling defects on proximal and mid left anterior descending artery; (**B**)—detailed angiographic view; (**C**)—OCT—longitudinal view of SRCT; (**D**)—OCT—3D reconstruction longitudinal view of SRCT; (**a**–**d**) OCT—transversal views of SRCT with no signs of atherosclerosis, with possible SCAD etiology. Conservative treatment was applied, due to the scarred myocardium.

The SRCT underlying mechanism is unknown, three possible origins being suggested: in situ dissection of an atherosclerotic plaque (Figure 6), post-spontaneous dissection (Figure 7) and cardio-embolic. [10].

### 6.3. Clinical Presentation

SRCT patients usually present with chest pain or pain equivalent in the setting of stable CAD or ACS, but can also be asymptomatic, as a concomitant, non-culprit lesion in ACS patients [10].

Studies on histopathologic specimens has shown that one third of acute thrombotic occlusions have some degree of recanalization [36].

The SRCT channels along with collaterals may play an important role in maintaining the viability of the subtended myocardium. The majority of these lesions remain functionally significant and require revascularization [39].

There are studies showing that recanalization can occur as fast as 10 days from the myocardial infarction onset.

SRCT may be the substrate of new acute coronary syndromes as many neovascular channels lack the proper connective tissue framework and are prone to rupture and in situ thrombosis [39]. SRCT was also discovered in a case of late intrastent restenosis [39].

### 6.4. Diagnosis of SRCT

As it was mentioned before, the true prevalence of SRCT lesions might be underestimated, due to its angiographic non-specific aspect: braided filling defect, haziness or pseudodissection, but can also appear as occlusions. This angiographic aspect can be related to other various conditions such as fresh thrombosis on plaque rupture/erosion, spontaneous dissection, aneurysm or heavy calcification [10]. Other authors suggested that the angiographic combination of braided and pseudodissection aspect is highly suggestive for SRCT [10]. SRCT lesions affects all coronary branches, without any typical prevalence. The diagnosis becomes possible only by intravascular imaging: OCT or IVUS.

#### OCT in SRCT

SRCT is a structure with multiple channels divided by smooth, high-luminosity, strong reflection and weak attenuation septa. Inside septa, signal-poor, darker tissue areas are visualized, linked by previous studies [10] to proteoglycan-rich tissue. Besides the crucial role of providing the SRCT diagnosis, the high-resolution OCT, offers very important information regarding the possible underlying pathological mechanism. In some cases, OCT can identify markers of atherosclerosis: lipid infiltration or intimal thickening and, also, possible markers of high-risk plaques such as: hypervascularization, calcium nodules, cholesterol crystals or inflammation, suggesting the complicated atherosclerotic plaque thrombus origin. In other cases, where there are no markers of atherosclerosis, a possible embolic or post-dissection thrombus origin can be suggested [10].

### 6.5. Treatment

Not all coronary lesions have indication for revascularization and this rule is applicable to SCRT lesions as well; that is why another very important role of OCT is lesion severity assessment. In one study [10], all SRCT lesions had borderline coronary angiography aspect, but significant OCT stenosis. OCT also identified longer lesion length, as compared to CA alone.

In SRCT cases, the discordance between lumen reduction and symptomatic impact are caused by particular SCRT spatial conformation namely tortuosity of the channels, translated into a greater hemodynamic impact of SRCT than that expected from stenosis severity [40].

OCT can also provide very important information regarding the process of selecting PCI materials and assessment of post PCI result. It is well known the fact that sub-optimal PCI result (malposition, edge dissection, under-expansion) is responsible for future MACCE, thus, the use of OCT for PCI guidance enriches the chances for a good immediate and late outcome [4].

SRCT has no particular treatment method. When indicated, PCI or surgery should be performed. In the literature, there were mentioned other treatment methods such as bioresorbable vascular scaffolds implantation, drug eluting balloon angioplasty and conservative treatment for the insignificant SRCT lesion [10].

### 6.6. Outcome and Prognosis

On a median follow-up of 29 months, patients with SRCT lesions, treated with DES implantation or medical therapy, were asymptomatic, in good clinical shape, with no major cardiovascular adverse events reported [10].

## 7. Conclusions

Non-atherosclerotic lesions such as SCAD and SCRT are less common in the clinical practice but have similar clinical manifestations as well as complications as the atherosclerotic lesions. The true prevalence of such lesions is underestimated, due to the unspecific angiographic aspect, more often in case of SRCT. The correct diagnosis can be made only by using intravascular imaging. OCT, with its high image resolution, is the perfect tool not only for the diagnosis, but for characterization and identification of pathogenic mechanisms, as well as for optimal treatment selection.

## Data Availability

The data presented in this study are available on request from the corresponding author. The data are not publicly available because they are property of Cluj County Emergency Hospital, Cluj-Napoca, Romania.
